# A Randomized Controlled Trial Evaluating the Levels of the Biomarkers hs-CRP, IL-6, and IL-8 in Patients with Temporomandibular Disorder Treated with LLLT, Traditional Conservative Treatment, and a Combination of Both

**DOI:** 10.3390/ijerph19158987

**Published:** 2022-07-23

**Authors:** Abdalwhab MA Zwiri, Wan Muhamad Amir W. Ahmad, Jawaad Ahmed Asif, Khoo Suan Phaik, Adam Husein, Nur Karyatee Kassim, Zuryati Ab-Ghani

**Affiliations:** 1 School of Dental Sciences, Health Campus, Universiti Sains Malaysia, Kubang Kerian 16150, Kelantan, Malaysia; wmamir@usm.my (W.M.A.W.A.); jawaad@usm.my (J.A.A.); adamkck@usm.my (A.H.); karyatee@usm.my (N.K.K.); 2 Hospital Universiti Sains Malaysia, Kubang Kerian 16150, Kelantan, Malaysia; 3 Department of Oral Diagnostic and Surgical Sciences, School of Dentistry, International Medical University, Bukit Jalil 57000, Kuala Lumpur, Malaysia; suanphaik_khoo@imu.edu.my

**Keywords:** TMD treatment, TMD biomarkers, LLLT, IL-6, IL-8, hs-CRP

## Abstract

Temporomandibular disorders (TMDs) are a type of idiopathic orofacial pain. Inflammation, particularly elevated circulating levels of high-sensitivity C-reactive protein (hs-CRP), interleukin-6 (IL-6), and interleukin-8 (IL-8), has been linked to pain symptoms. The purpose of this study was to compare hs-CRP, IL-6, and IL-8 biomarkers and pain intensity with different treatment strategies (LLLT, standard conservative treatment, and combination) for TMD patients. Methods: A total of 32 participants were randomly included in the study and divided into three groups (Group I, Group II, and Group III) referred from the Dental Clinic, School of Dental Science, HUSM. Patients received LLLT (Groups II and III) in five sessions for the duration of 10 days. Patients in Groups I and III received standard conservative TMD treatment (diet and stress counseling, jaw exercises, physical therapy, which was a hot towel application) by the principal investigator. All blood samples for biomarkers were performed before starting treatments and directly after finishing the treatment protocols, where all results were recorded. Results: The result showed a significant difference in the mean IL-8 (*p* = 0.001) between the three intervention groups (LLLT, standard treatment, and combined treatment). IL-6 showed an increase in the mean of IL-6 levels from baseline to post-treatment with a better mean in the LLLT treatment group without any significant differences. Additionally, there were no significant mean differences found between the groups and in the group for the hs-CRP biomarker. Conclusions: A statistically non-significant difference was found in hs-CRP and IL-6 before and after LLLT, conservative, and combined treatment strategies of TMD. A statistically significant difference was observed in the mean levels of IL-8 between the LLLT intervention group and the combined treatment group. Although there was no statistically significant correlation between pain intensity and biomarkers, a statistically significant difference was found in pain intensity before and after LLLT, conservative, and combined treatment strategies. TMJ degeneration could be exacerbated by elevated IL-8 levels. Thus, this can be an important biomarker to mark or identify the painful condition of TMJ.

## 1. Introduction

The pain in the orofacial region is the primary complaint for which patients seek treatment. One condition that possesses a significant global health problem is temporomandibular disorder (TMD), according to the American Academy of Pain Management (AAP) and the American College of Surgeons (ACSP). The temporomandibular joint disease (TMD) affects about 5–12% of the general population. TMD is defined as a group of clinical conditions that affect the temporomandibular joint (TMJ) with related structures and the masticatory muscles. Women have a higher risk of developing TMDs than men, with some studies reporting a 25–40% increase [1]. It is widely accepted that the cause of TMD is a combination of factors, including biomechanical, neuromuscular, biopsychosocial, and biological factors [2,3]. The temporomandibular joint’s (TMJ) molecular complexity makes it difficult to identify the distinct causes of TMJ disorders (TMDs) and, consequently, develop a therapy. The balance between free radical reduction and free radical creation may be altered, accelerating the progression of a diseased joint [4]. Furthermore, the recognition of the relationship between TMDs and increased levels of biochemical or inflammatory markers allows for the exploration of more sensitive and new biomarkers in this area [1].

Biomarker research has grown in popularity in recent years as a potential diagnostic method. The FDA defines biomarkers as “a defined characteristic that is objectively measured as an indicator of a normal biological process, a pathologic process, or biological responses to a therapeutic intervention.” One of the most important aspects of biomarker research is its ability to predict the patient’s pain state’s onset, duration, severity, and prognosis [5]. Although many synovial, serum, and urinary proteins demonstrate useful diagnostic value for TMD, no perfect and direct package of disease markers for TMDs is being used as a daily activity in medical practice. However, there is sufficient progress being made, which demonstrates the attempts to perform a detailed assessment of this area and future directions of research based on the accumulated evidence [6]. Cytokines are the crucial polypeptide moderators of serious and severe inflammatory processes [7]. Monocytes and macrophages infiltrate the synovium and release cytokines, which alter the viscosity of the synovial fluid and reduce the lubrication and nutrition of articular cartilage and disks. Proteinases, which deplete cartilage’s proteoglycans, are stimulated by cytokine production and released during inflammation [4]. In general, studies showed that the most likely possible TMD biomarkers were IL-6, IL-8, IL-1, and TNF [6]. Furthermore, the level of C-reactive protein (CRP) in TMD patients with chronic pain was also evaluated [8]. Interestingly, in another very recent study aimed to assess the effectiveness of phonophoresis in patients with TMD, researchers used hs-CRP to assess inflammation before and after treatment and reported that hs-CRP can be used as an indicator of progressive TMJ inflammation [6]. However, inconsistent literature findings have been recorded even when the same test methods were used. Biomarkers’ diagnostic and prognostic abilities cannot be compared because of these findings. Despite the importance of the diagnostic process’s biomarker profile for TMD patients, further research is needed to identify gold-standard biomarkers [1,6].

Conservative “reversible” management of TMD remains the most common approach to the management of more than 90% of patients [9]. Conservative treatment methods for TMDs include psychological [10,11], splints [9,12], pharmacological therapies [13,14], physiotherapy [15,16], self-management [17], and low-level laser therapy (LLLT) [18,19,20]. Irreversible treatment involves orthodontics, occlusal adjustments [21,22], and surgery [14]. A key component of primary non-invasive treatment is known as “self-care” or “self-management” (SM). Self-care or SM can be all that is needed for people who are responsive or enthusiastic or can be part of more complicated approaches [23,24]. Such SM techniques are often of primary importance in providing patients with some control in monitoring their symptoms in chronic TMD attacks or flare-ups, in addition to initial treatment [17]. In a recent systematic review [25] that looked at the effectiveness of SM approaches, it was reported that SM is not only low-tech and non-invasive but also successful in reducing pain and enhancing functions. However, the efficacy of SM as a stand-alone treatment was not yet known, as it was typically the comparator treatment and was not correlated with any treatment or control groups [24]. Therefore, further research is needed to confirm that SM programs are more beneficial than no treatment and/or placebo at all. With more standardized SM programs, greater clinical results could be achieved, and it would be easier for clinicians to repeat successful interventions if SM programs reported in the literature describe their modules with the use of established behavioral change categorization [26]. Among all non-surgical treatments for TMD, LLLT has recently been put under the spotlight because its proponents claim that this approach has easy application, limited treatment time, and minimum contraindications [18]. LLLT was used to alleviate the signs and symptoms of TMD patients based on its biostimulative, regenerative, analgesic, and anti-inflammatory effects. However, in order to draw firm conclusions, it is necessary to standardize the application criteria, such as the type of TMD and the parameters such as intensity and frequency [18]. Only time will tell whether or not LLLT is effective in treating TMD. There are too many methodological variations among the studies to provide uniform guidelines for effective LLLT treatment [19,20].

Therefore, this study aimed to compare the effect of different treatment strategies (LLLT, standard conservative treatment, and combination) on TMD patient biomarkers (IL-6 and IL-8, hs-CRP) and pain intensity before and after receiving treatment.

## 2. Materials and Methods

### 2.1. Study Design

This study was a randomized controlled trial (RCT) study with ACTRN (ACTRN12620000442909). Patients with complaints of TMD pain, who came to the Dental Clinic at the Hospital Universiti Sains Malaysia were selected for this study. Ethical approval was obtained from the Human Research Ethics Committee USM (HREC) with the reference number of USM/JEPeM/19010088.

### 2.2. Patient Selection

**Figure 1:**
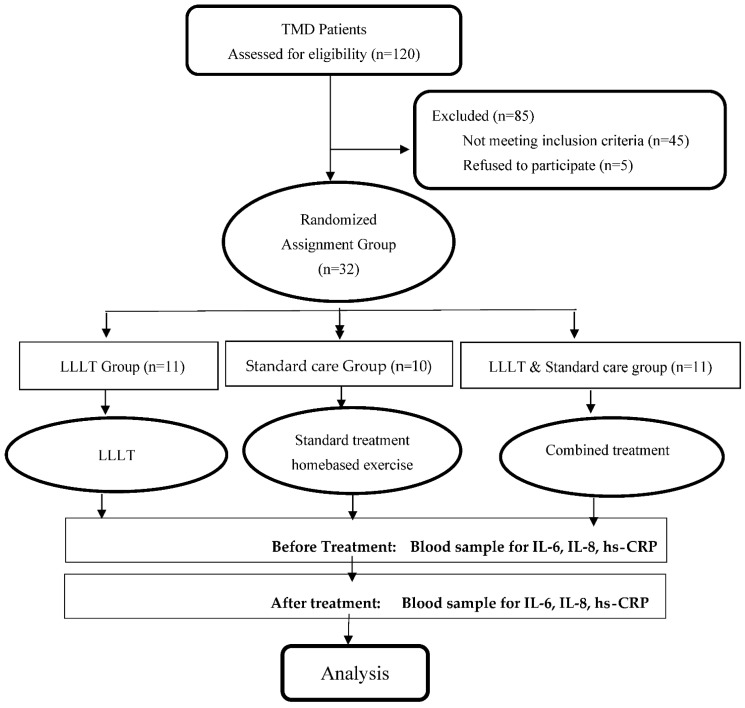
Subject recruitment and data collection flowchart.

The participants of this study consisted of a total of 32 individuals divided into 3 groups: Group I (conservative treatment group, *n* = 10), Group II (low-level laser therapy (LLLT) only, *n* = 11), and Group III (combined treatment group, *n* = 11) (Figure 1). The sample size was calculated for each objective based on the analysis of pain variability (visual analogue scale (VAS)) as the primary outcome measure in TMD studies by using PS software, where power is 0.8, and type I error is 0.5, with a mean difference of 21.2 and SD 14.4 to study the effect of standard conservative treatment [27]; a mean difference of 2.9 and SD 1.89 to study the effect of LLLT [28]; and a mean difference of 2.5 and SD 1.64for the study of the effect of combined treatment [29]. Participants were patients referred from the Dental Clinic, School of Dental Science, HUSM. As patients may have more than one type diagnosed with RDC/TMD criteria, those with mixed types of TMDs (atherogenic and myogenic) were included in this study, as well as those aged 18 years and older and having good general health conditions. The patients with trauma, craniofacial pathology, and previous TMJ surgeries, any chronic systemic diseases, e.g., diabetes mellitus, were excluded from this study. Those patients who were taking antibiotics within the last 2 weeks before the start of the study, had previously undergone LLLT treatment, or were taking immunosuppressant drugs and aspirin were also excluded from this study. Pregnant and lactating women were not included in this study. Informed consent was obtained from all the patients. The common clinical symptoms of the patients were chronic pain, limited mouth opening, mouth soreness, jaw discomfort, masticatory muscle tenderness, headache, clicking, crepitus, tinnitus ear pain, etc. Patients were randomized, after initial screening, and the treatment plan was implemented with the final diagnosis, with the patients providing free and voluntary consent before providing written informed consent. Microsoft Excel software was used to generate a random number from 1 to 10, and the blocks were assigned to each number accordingly. The generated blocks were copied, and each alphabet of the block was written down and sealed separately in an envelope according to their sequence.

### 2.3. Data Collection Procedure

#### 2.3.1. Intervention

In Groups II and III, patients received five sessions of low-level laser treatment every other day for a total of 10 days (0, 2nd, 4th, 6th, 8th, and 10th day). In this study, an Ezlase 940 Diode Laser (USA), a Class IV GaAlAs, InGaAsP diode laser therapy system was utilized, following standard protocol [30,31]. Regarding treatment for TMD, patients in Groups I and III were given the same standard conservative TMD therapy (diet and stress counseling, jaw exercises, and physical therapy using hot towels) by the study’s principal researcher [24]. Each patient underwent two 30 min face-to-face training sessions on written instructions, one before treatment began and the other two weeks later, in a total of four weeks of treatment. It was possible to reverse the effects of conservative therapy, which was non-invasive, reversible, and patient-centered. At each follow-up appointment, patients filled out a control card (checklist) detailing the techniques and exercises they had been practicing at home, and this card was given back to the doctor to verify their continued compliance with the treatment plan. The outcome measurement was evaluated before the commencement of treatment (blood sample for hs-CRP, IL-6, and IL-8) and immediately after the end of each treatment (blood sample for hs-CRP, IL-6, and IL-8.

#### 2.3.2. Biomarker’s Assay, Serum Interleukins IL-6, IL-8, and hs-CRP

Venipuncture safety precautions were used to obtain all of the blood samples. There were only two times that 5 mL of blood was taken from each subject in a plain tube: once before the start of treatments and once after the end of treatments; both were sent to the laboratory for hs-CRP, IL-6, and IL-8 analysis. Following an 8 min centrifugation at 3000 rpm, the samples were kept at −80 °C until further testing. An Elecsys IL-6 immunoassay reagent kit (Roche Diagnostics, Rotkreuz, Switzerland) was used for the quantitative determination of IL-6 in collected human serum in this study. A high-sensitivity C-reactive protein (Latex) immunoassay kit (Roche Diagnostics, Switzerland) was used for the quantitative determination of hs-CRP in collected human serum in this study. An Elabscience commercial ELISA kit was used to measure the levels of IL-8 in the serum, which was processed using a COBAS 6000 analyzer (Roche Diagnostics, Switzerland). Biomarker testing was performed directly before and after treatment protocols were finished for each group. As the patients were followed up for a long period for clinical symptoms, biomarkers were measured only twice (before and directly after the end of each treatment protocol in each group, before and after LLLT for Group II, before and after conservative treatment for Group I, and before and after combined treatment for Group III) to avoid obtaining a biased result pointing to other systemic diseases or any inflammatory response during the course; all results were recorded.

### 2.4. Statistical Analysis

The obtained data were analyzed by using IBM SPSS (Statistical Package for the Social Sciences) version 25.0. For descriptive analysis, the obtained data were analyzed with continuous variables and categorical variables, which were exhibited in mean, standard deviation (SD), and various clinical parameters. Between groups, repeated-measures ANOVA was also performed to determine the group effect and the interaction between groups, and the time effect. Pearson’s correlation analysis was performed to determine the association between the clinical biomarkers and pain outcome. The level of statistical significance was set at *p* < 0.05.

## 3. Results

**Figure 2:**
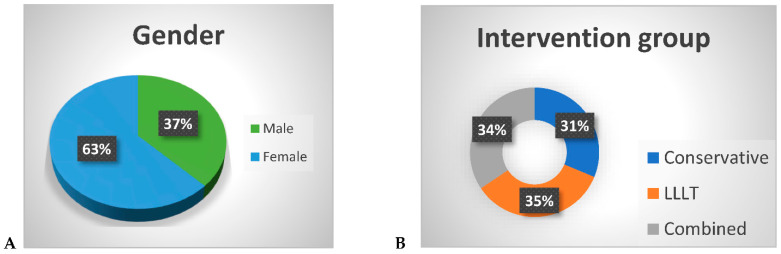
Sociodemographic characteristics of the patients: (**A**) gender; (**B**) intervention group.

**Figure 3:**
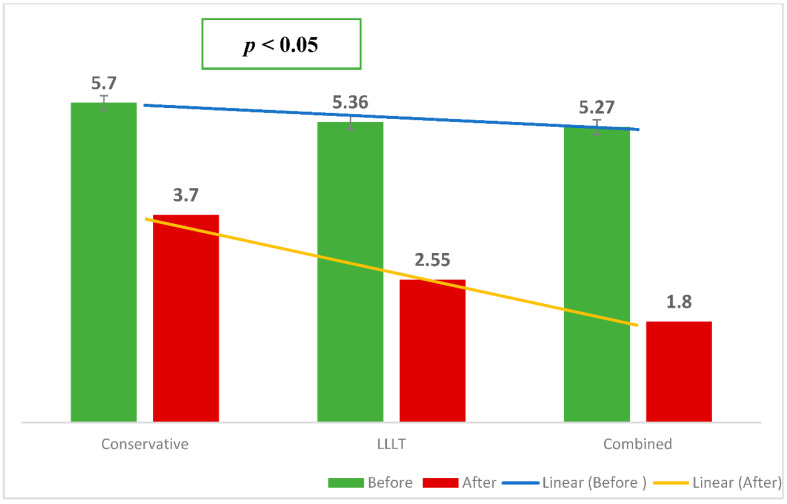
Effectiveness of the intervention group in terms of pain intensity score.

Figure 2 shows the sociodemographic characteristics of the patients participating in this study. A total of 32 patients we recruited for this study (mean age = 20.9, SD = 10.41). There were 12 males (37.5%) and 20 females (62.5%). The participants were randomly allocated to three treatment groups: home-based therapy (10), LLLT (11), and combined treatment (11). The combined group and LLLT treatment group proved more effective than the standard group in terms of reducing the pain intensity of the TMD patients (*p* < 0.05) (Figure 3).

### 3.1. Effect of the Treatments (LLLT, Standard Treatment, and Combined Treatment) on Interleukin 6 (IL-6) Biomarkers

#### 3.1.1. Within-Group Measures’ Analysis (Time Effect Regardless of Group)

The effect of the treatment interval time on IL-6 is shown in Table 1. There was no statistical significance in the mean levels of IL-6 before and after treatment over time (*p* = 0.920), although there was an increase in the mean of IL-6 levels from baseline to post-treatment.

#### 3.1.2. Between-Group Analysis (Group Effect Regardless of Time)

Table 1 shows a statistically significant difference in the mean levels of IL-6 across the three treatment groups (LLLT, standard treatment, and combined treatment), *p* = 0.037, for the effect of treatment groups on IL-6. Therefore, multiple pairwise comparisons were made with α correction using the Bonferroni correction method. The pairwise comparison showed that all the pairs were not statistically significant: the combined treatment group had the highest mean at the baseline, while the LLLT treatment group had the highest mean post-treatment.

#### 3.1.3. Interaction Effect (Time Effect × Group Effect)

**Figure 4:**
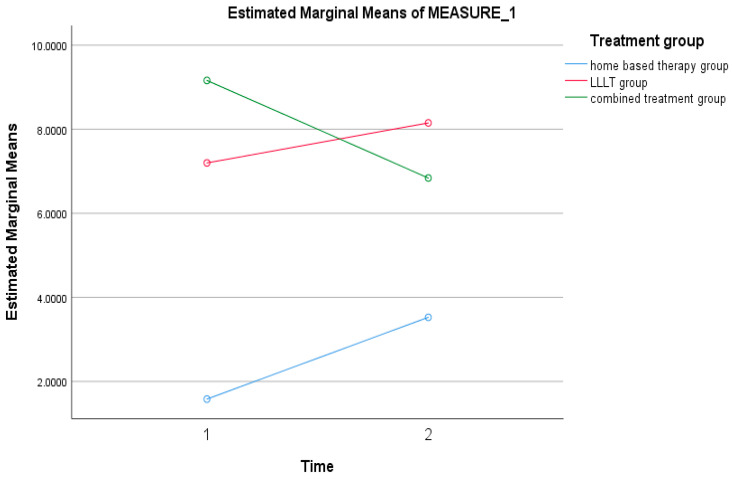
Trend of mean IL-6 for home-based therapy, LLLT, and combined therapy groups.

There was no statistical significance in the difference between the mean levels of IL-6 before and after treatment over time (*p* = 0.629) (Table 1 and Figure 4).

### 3.2. Effect of the Treatments (LLLT, Standard Treatment, and Combined Treatment) on Interleukin 8 (IL-8) Biomarkers

#### 3.2.1. Within-Group Measures’ Analysis (Time Effect Regardless of Group)

For the effect of the treatment interval time on IL-8, the results revealed that there was no statistical significance in the difference between the mean levels of IL-8 before and after treatment over time, although the mean was higher at post-treatment (*p* = 0.695) (Table 2).

#### 3.2.2. Between-Group Analysis (Group Effect Regardless of Time)

For the effect of the treatment group on IL-8, the results revealed that there was a statistically significant difference in the mean levels of IL-8 between the three treatment groups (LLLT, standard treatment, and combined treatment), *p* = 0.001. Therefore, multiple pairwise comparisons were made with α correction using the Bonferroni correction method. The pairwise comparison showed a statistically significant difference in the mean levels of IL-8 between the LLLT and combined groups only, *p* = 0.001 (Table 2).

#### 3.2.3. Interaction Effect (Time Effect × Group Effect)

**Figure 5:**
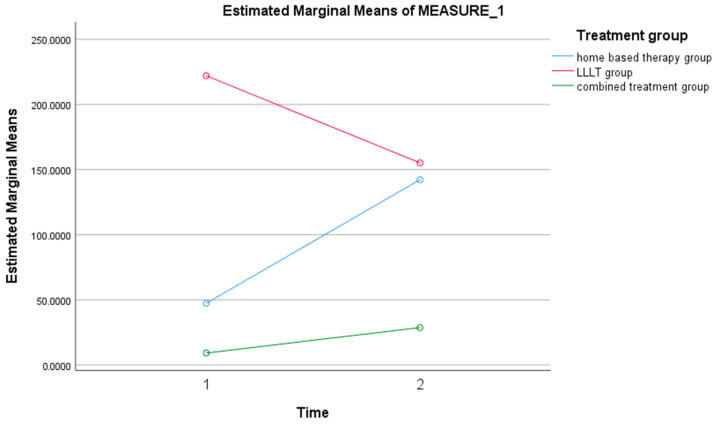
Trend of mean IL-8 for Home based therapy, LLLT, and combined therapy groups.

Table 2 shows that there was no statistically significant interaction between the time effect and the group effect. This indicated that there was no statistically significant difference in the mean levels of IL-8 before and after treatment for all the treatment groups (*p* > 0.05). Nonetheless, at the baseline, the LLLT had the highest mean, which was significantly higher than the mean for the home-based group. Additionally, at post-treatment, the mean levels of IL-8 were highest in the LLLT treatment group, although there was no statistically significant difference in the mean levels of IL-8 among the other treatment groups (Figure 5).

### 3.3. Effect of the Treatments (LLLT, Standard Treatment, and Combined Treatment) on High-Sensitivity C-Reactive Protein (hs-CRP) Biomarkers

#### 3.3.1. Within-Group Measures Analysis (Time Effect Regardless of Group)

Table 3 shows that there was no significant difference between the mean levels of hs-CRP before and after treatment with time, *p* = 0.714. However, the mean was higher post-treatment.

#### 3.3.2. Between-Group Analysis (Group Effect Regardless of Time)

For the effect of treatment group on hs-CRP, the results revealed that there was no significant difference of the mean level of hs-CRP between the three treatment groups (LLLT, standard treatment, and combined treatment) *p* = 0.490. The combined treatment group has the highest mean at baseline and post treatment (Table 3).

#### 3.3.3. Interaction Effect (Time Effect × Group Effect)

**Figure 6:**
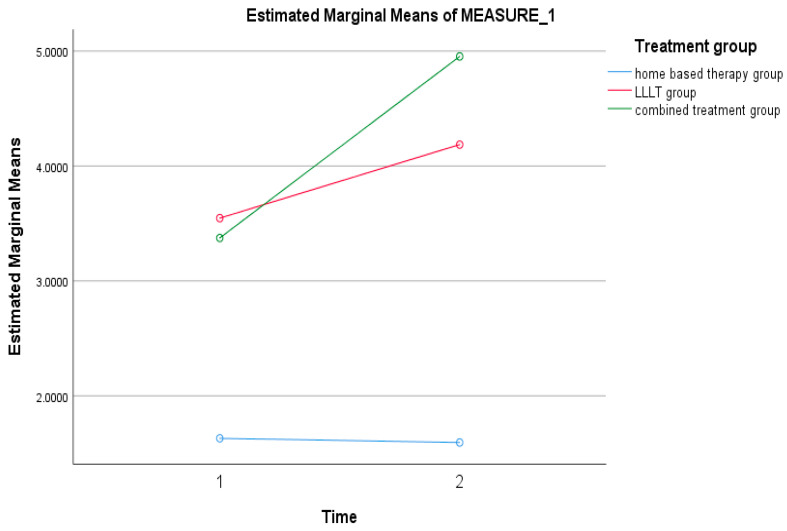
Trend of mean hs-CRP for home-based therapy, LLLT, and combined therapy groups.

Based on the findings of this study, there was no significant interaction between the time effect and the group effect, *p* = 0.945. This indicated that there was no significant difference between the mean levels of hs-CRP before and after treatment for all the treatment groups (Table 3 and Figure 6).

### 3.4. Correlation between the Clinical Biomarkers and Pain Outcomes

Pearson’s correlation analysis was performed to determine the association between the clinical biomarkers and pain outcomes (Table 4).

## 4. Discussion

### 4.1. Effects of the Treatment Groups (LLLT, Standard Treatment, and Combined Treatment) on Interleukin 6 (IL-6)

TMJ issues affect millions of people around the world, and they are characterized by discomfort and joint dysfunction. Increased pressures in the TMJ are caused by masticatory muscle stimulation caused by malocclusion, physical stress, anxiety, and oral habits. The forces that are transmitted to the TMJ structures during clenching and jaw movements are compressive and tangential in character [32]. IL-6 has been identified as one of the most important proinflammatory cytokines in the etiology of TMJ with internal derangement (ID) [6,7]. The IL-6 gene is located on chromosome 7. IL-6 is a glycopeptide with a molecular weight of 26 kDa that is produced by a range of cell types, including fibroblasts, osteoblasts, endothelial cells, monocytes, keratinocytes, T cells, and B cells. IL-6 works via two distinct signaling pathways: classic signaling via the membrane-bound IL-6 receptor (mIL-6R) through glycoprotein gp130 activation, or trans-signaling via soluble receptors (sIL-6R). There is substantial evidence that classic signaling plays a role in tissue regeneration, while trans-signaling is responsible for the vast majority of proinflammatory responses [33]. In the present study, the result showed no statistical significance in the difference between the mean levels of IL-6 before and after treatment with time. However, in the three intervention groups (LLLT, standard treatment, and combined treatment), there was a difference in the mean levels of IL-6, which was statistically significant (*p* = 0.037). The mean levels of IL-6 at baseline and 12 weeks were 5.980 ± 1.495 µg and 6.171 ± 1.082 µg, respectively. Therefore, patients who received treatment for a TMD regardless of its modality (except for those in the combined treatment group) showed an increased level of IL-6. At baseline, patients in the combined treatment group had the highest mean level of IL-6 (9.16 ± 12.80 µg, which then decreased after 12 weeks of treatment to 6.84 ± 5.659 µg). Therefore, it can be concluded that IL-6 levels increased in TMD patients who received home-based and combined treatment but decreased after receiving the LLLT alone. Similarly, in a study conducted by Baş et al., (2019) comparing groups of the occlusal splint and home-based therapy, a significant symptomatic improvement was observed after treatment (*p*  <  0.005). The patients’ major symptoms were pain in the TMJ area and limited mouth opening. No statistically significant differences were found between the two groups concerning pre-treatment and 3-month levels of IL-6. However, therapy was found to be successful in eliminating clinical symptoms of TMDs [34]. Shinoda and Takaku (2000) reported that IL-6 levels in the TMJ aspirates of patients with chronic TMJ disorders were raised. Therefore, this indicates that IL-6 may be involved in the development of persistent TMJ problems [35], and therefore, monitoring the levels of IL-6 will be of value in charting the patient’s treatment progress.

Furthermore, interleukin-6 (IL-6) is a proinflammatory cytokine that stimulates the hypothalamic–pituitary–adrenocortical (HPA) axis by inducing the release of corticotrophin-releasing hormone (CRH) from the paraventricular nucleus. Stressful occurrences have been linked to increased symptomatology in TMD patients. In 2002, Costello et al. reported an association between stress and IL-6 in TMD patients whose levels of IL-6 significantly reduced (β = 0.31) after having corrected their depressed moods, compared with controls. These findings suggest that TMD patients may have a reduced adrenergic response to the challenge, possibly due to alterations in receptor responsivity associated with long-term increases in a sympathetic drive (adrenergic receptor downregulation). These reductions in sympathetic response may facilitate a limited response to inflammatory cytokines such as IL-6 [36]. The results of the present study showed a significant difference in the mean levels of IL-6 among the three different treatment groups, which indicates that controlling stress and anxiety as well as depression is beneficial for achieving a successful treatment outcome in TMD patients.

In contrast to hormones, cytokines act locally and respond mainly to cellular stresses. Therefore, local stresses to the synovial tissues of the TMJ can produce proinflammatory cytokines such as IL-6, which clinically manifest as pain and dysfunction of mandibular movement [37]. The results of the present study indicated that LLLT had a significant role in a reduction in IL-6 inflammatory markers. This concurs with the results of Wang et al., (2015), who showed that laser therapy in conjunction with aerobic training may provide a therapeutic approach for reducing inflammatory markers (IL-6 and TNF-α). However, LLLT alone without exercise was not able to improve physical performance [38].

### 4.2. Effects of the Treatment Groups (LLLT, Standard Treatment, and Combined Treatment) on Interleukin 8 (IL-8)

IL-8 is a proinflammatory cytokine that has been linked to the regulation of cartilage degradation and bone remodeling, including bone resorption [39]. It was detected in 80% of the synovial tissue specimens taken from the TMJs with inflammatory disease (ID). However, IL-8 does not exist in healthy TMJs [40]. Instead, IL-8 is a cytokine produced by blood cells, endothelial cells, and fibroblasts that acts as a mediator in the inflammatory response. Levels of IL-8 are found to be higher in patients with persistent back pain, fibromyalgia, chronic fatigue syndrome, and TMDs [41]. A study showed that IL-8 levels were raised during intramuscular microdialysis in TMD patients with jaw muscle pain in comparison to controls [42]. The present study showed there was a statistically significant difference in the mean levels of IL-8 among the three treatment groups (*p* = 0.001). However, when the mean levels of IL-8 were measured before and after treatment for each of the respective groups, there was an increase in the mean levels post-treatment, but these were not statistically significant. Another study by Cê et al., (2018) found a significant difference in IL-8 levels in TMD patients, compared with those of healthy individuals. The study reported that patients with TMDs had considerably higher salivary IL-8 levels. Increased IL-8 levels suggest that they could be one of the causes of TMJ degeneration, leading to a decrease in the TMJ’s adaptive capability [43].

Another study conducted by Kacena et al., (2001) showed that the levels of proinflammatory cytokines such as TNF-, IL-1ß, IL-6, IL-8, and IFN-Á were markedly increased in patients with TMD, compared with healthy patients. The fact that proinflammatory cytokines were increased in the synovial fluid of TMD patients indicates that proinflammatory cytokines are generally associated (causative) with the regulation of acute and chronic inflammation and connective tissue destruction. One of the more relevant mechanisms induced by the proinflammatory cytokines in the chronic inflammation seen in TMDs is the release of collagenase and PGE2 [44]. In this present study, among the three treatment groups, patients receiving LLLT treatment showed a reduction in the mean levels of IL-8 post-treatment. The mean value of IL-8 was 222.11 ± 186.351 IU at baseline and 155.19 ± 222.940 IU after 12 weeks of treatment. This significant reduction indicates that LLLT is a good treatment option for the management of TMD. Though the home-based and combined treatment groups showed an increase in the mean levels of IL-8 after treatment, the patients in the home-based treatment group showed the highest mean level (142.23 ± 197.477 IU). The results of this study indicate that home-based treatment on its own is not beneficial in the management of TMD, especially in terms of reducing the levels of IL-8.

IL-8 is a chemokine that promotes chemotaxis and the activation of neutrophils and is known to cause neutrophil infiltration into the synovial fluid and enhance joint inflammation in rheumatoid arthritis. As the TMJs are inflamed in TMD patients, IL-8 may have a role in the production of inflammatory cells in the TMJ [40]. Therefore, the presence of IL8 or its increased levels (e.g., post-treatment) is an indication of the production of pathological conditions including TMJ degeneration. According to Núñez et al., LLLT, with the appropriate parameters, may act as an analgesic and anti-inflammatory mediator in promoting muscle relaxation. LLLT may provide analgesic benefits through a variety of pathways (e.g., increases endogenous opiate liberation, lowers nerve cell membrane permeability, decreases analgesic drug release in diseased areas, increases ATP generation, and decreases tissue asphyxia) [45]. The results of the present study showed that patients receiving LLLT had improved parameters post-treatment and showed the best treatment outcomes among the three treatment groups.

### 4.3. Effect of the Treatment Groups (LLLT, Standard Treatment, and Combined Treatment) on High-Sensitivity C-Reactive Protein (hs-CRP)

CRP is a new and reliable biomarker of the acute phase response to infectious burdens and/or inflammation. hs-CRP levels have been observed to be high in a variety of disorders, including periodontal disease, gangrenous pulp, fungal diseases of the prosthetic base, and post-traumatic situations such as jaw fractures. Due to its kinetics, it best depicts the individual’s inflammatory condition [46,47]. In 2004, D’Aiuto et al. conducted a pilot study on 94 subjects and assessed serum hs-CRP and IL-6 levels at baseline and 2 and 6 months following non-surgical periodontal therapy. They found a significant reduction in hs-CRP and IL-6 serum levels, along with improvement in all clinical periodontal parameters with therapy [48]. The results of the present study showed that the mean levels of hs-CRP were higher post-treatment, but these were not statistically significant. The increased level of hs-CRP indicated that three of the treatment modalities did not show any statistically significant correlation with the disease condition. However, the home-based treatment showed a decreased mean value of hs-CRP 1.59 ± 2.358 IU directly after. Similar results were reported by a Japanese study that showed no statistically significant difference in IL-6 and hs-CRP before and after therapy. The authors of that study suggested that the lack of statistical significance may be due to the varying contributions of periodontal disease to the total burden of inflammation in different patients, as well as the relatively small number of patients [49]. As a result, the fact that hs-CRP can be changed by any inflammatory activity in the body could be the reason why there was not a significant difference between the therapy groups and time. Even though patients who received LLLT showed a better clinical response, with a significant difference in the levels of IL-6 and IL-8 but not for hs-CRP, this could be because hs-CRP is a general inflammatory marker, which means that it might have been affected by other inflammatory processes in the body while the trial was being conducted. Another study that was conducted by Ramakrishnan and Aswath (2019) to determine the efficacy of phonophoresis in patients with TMDs showed a significant difference in the VAS scores and hs-CRP levels before and after treatment, which is an indication of improvement in the TMJ inflammation. This study was conducted in order to determine the efficacy of phonophoresis in patients with TMD. According to the findings of this study, hs-CRP is a sensitive indicator of inflammation [50].

The result of the present study showed no significant correlation between pain intensity and biomarkers level except for IL6 at baseline and VAS directly after treatment (*p*-value < 0.05); this result is in accordance with the results of previous studies that found hs-CRP levels in TMD patients to be within normal levels, and the intensity of pain is probably not directly related to inflammation [47] or showed no statistically significant difference in IL-6 and hs-CRP before and after therapy [49]. This lack of statistical significance and correlation between pain intensity and biomarkers may be due to the varying contributions of TMDs to the total burden of inflammation in different patients. Moreover, it may be due to the fact that biomarkers are analyzed from blood serum, not from TMJ fluids, which makes it more prone to the general inflammatory status of the body.

Despite the fact that our findings are similar to those of other studies, this study was conducted in a single center (USM). It excluded surgical treatment of TMJ disorders as well as some age groups, especially children and adolescents (below 18 years). We tried to follow an acceptable statistical sample size for this study with full protocols. A systematic review and metanalysis of similar topics also reported that most of the clinical studies conducted concerning TMDs recruited around 36 subjects [6]. Proper double blinding was not achieved due to the exposure of the treatment to the patients except during block distribution.

## 5. Conclusions

Although there was no statistically significant correlation between pain intensity and biomarkers, statistically significant differences were found in pain intensity before and after LLLT, conservative, and combined treatment strategies. A statistically non-significant difference was noticed in IL-6 before and after LLLT, conservative, and combined treatment of TMD. There was a statistically significant difference in the mean levels of IL-8 between the LLLT intervention group and the combined treatment group. No significant difference was observed between the mean levels of hs-CRP before and after treatment for all treatment groups. TMJ degeneration could be exacerbated by elevated IL-8 levels. Thus, this can be an important biomarker to mark or identify the painful condition of TMJ.

## Figures and Tables

**Table 1:** Effect of time and treatment groups on IL-6.

Variable	Mean (SD) pg/100 mL	F-Statistic (df)	*p*-Value
**IL-6 overall score**		0.010 (1, 29)	0.920
IL-6 at baseline	5.98(1.50)		
IL-6 directly after treatment	6.17 (1.08)		
**Between treatment group**		3.686 (2, 29)	0.037
**Time vs. treatment group**		0.471 (2, 29)	0.629
IL-6 at baseline *			
Home-based	1.58 (0.08)	2.247 (2, 29)	0.124
LLLT	7.20 (6.56)		
Combined treatment	9.16 (12.80)		
IL-6 directly after treatment *			
Home-based	3.53 (2.73)	1.576 (2, 29)	0.224
LLLT	8.15 (8.35)		
Combined treatment	6.84 (5.66)		

Box test of equality of covariance matrices: 85.67 (12.85); *p*-value < 0.001. * = multiple pairwise comparison.

**Table 2:** Effect of time and treatment groups on IL-8.

Variable	Mean(SD) pg/100 mL	F-Statistic (df)	*p*-Value
**IL-8 overall score**		0.157 (1, 29)	0.695
IL-8 at baseline	92.83 (20.63)		
IL-8 directly after treatment	108.69 (30.57)		
**Between treatment group**		8.844 (2, 29)	0.001
**Time vs. treatment group**		1.354 (2, 29)	0.274
IL-8 at baseline *			
Home-based	47.35 (71.30)	10.359 (2, 29)	<0.001
LLLT	222.11 (186.35)		
Combined treatment	9.13 (10.24)		
IL-8 directly after treatment *			
Home-based	142.23 (197.48)	1.767 (2, 29)	0.189
LLLT	155.19 (222.94)		
Combined treatment	28.65 (41.71)		

Box test of equality of covariance matrices: 85.67 (12.85); *p*-value < 0.001. * = multiple pairwise comparison.

**Table 3:** Effect of time and treatment groups on hs-CRP.

Variable	Mean (SD) mg/dL	F-Statistic (df)	*p*-Value
**hs-CRP overall score**		0.137 (1, 29)	0.714
CRP at baseline	2.85 (1.13)		
hs-CRP directly after treatment	3.58 (1.54)		
**Time vs. Treatment group**		0.056 (2, 29)	0.945
**hs-** **CRP at baseline**			
Home based	1.63 (2.33)	0.286 (2, 29)	0.753
LLLT	3.55 (9.14)		
Combined treatment	3.37 (5.40)		
**hs-** **CRP directly after treatment**			
Home based	1.59 (2.36)	0.422 (2, 29)	0.660
LLLT	4.19 (9.72)		
Combined treatment	4.95 (10.99)		

Box test of equality of covariance matrices: 36.50 (5.47); *p*-value < 0.001.

**Table 4:** Pearson’s correlation analysis between the clinical biomarkers and pain outcomes.

Biomarkers	VAS Baseline	VAS Directly after Treatment	VAS (4 Weeks)	VAS (8 Weeks)	VAS (12 Weeks)
IL-6 baseline	−0.08	0.36 *	−0.23	−0.25	−0.12
IL-6 post-tretament	0.04	−0.09	−0.01	0.04	−0.10
IL-8 baseline	0.11	−0.10	0.13	0.12	0.13
IL-8 post-treatment	−0.11	0.17	0.27	0.11	0.14
hs-CRP baseline	0.15	0.03	0.05	−0.03	−0.14
hs-CRP posttreatment	−0.12	−0.22	−0.30	−0.19	−0.13

* *p* < 0.05. There was a significant correlation between IL6 baseline and VAS directly after treatment (*p*-value < 0.05).

## Data Availability

Data are available upon request.
